# Perioperative fluid management and associated complications in children receiving kidney transplants in the UK

**DOI:** 10.1007/s00467-022-05690-3

**Published:** 2022-08-16

**Authors:** Natalie Wyatt, Karen Norman, Kate Ryan, Mohan Shenoy, Michal Malina, Lasanthi Weerassoriya, Jack Merritt, Ramnath Balasubramanian, Wesley Hayes

**Affiliations:** 1 Department of Paediatric Nephrology, Great Ormond Street Hospital for Children NHS Foundation Trust, Great Ormond Street, London, WC1N 3JH UK; 2 Department of Paediatric Nephrology, Nottingham Children’s Hospital, Nottingham, NG7 2UH UK; 3 Department of Paediatric Nephrology, Royal Manchester Children’s Hospital, Oxford Road, Manchester, M13 9WL UK; 4 Department of Paediatric Nephrology, Great North Children’s Hospital, Royal Victoria Infirmary, Newcastle upon Tyne, NE1 4LP UK; 5 Translation and Clinical Research Institute, Newcastle University, Newcastle upon Tyne, UK; 6 Department of Paediatric Nephrology, Birmingham Children’s Hospital, Steelhouse Lane, Birmingham, B4 6NH UK; 7 Institute of Child Health, University College London, London, UK

**Keywords:** Paediatric kidney transplant, Fluid, Graft survival, Pulmonary oedema, Hypertension, Oxygen requirement, PICU

## Abstract

**Background:**

Intravenous fluid administration is an essential part of perioperative care for children receiving a kidney transplant. There is a paucity of evidence to guide optimal perioperative fluid management. This study aimed to identify the volume of perioperative fluids administered across 5 UK paediatric kidney transplant centres and explore associations between fluid volume administered, graft function, and fluid-related adverse events.

**Methods:**

Data were collected from five UK paediatric kidney transplant centres on perioperative fluid volumes administered, and incidence of pulmonary oedema, systemic hypertension, and requirement for intensive care support. Children < 18 years of age who received a kidney-only transplant between 1^st^ January 2020 and 31^st^ December 2021 were included.

**Results:**

Complete data from 102 children were analysed. The median total volume of fluid administered in 72 h was 377 ml/kg (IQR 149 ml/kg) with a high degree of variability. A negative relationship between total fluid volume administered and day 7 eGFR was noted (*p* < 0.001). Association between urine volume post-transplant and day 7 eGFR was also negative (*p* < 0.001). Adverse events were frequent but no significant difference was found in the fluid volume administered to those who developed an adverse event, vs those who did not.

**Conclusions:**

This study describes a high degree of variability in perioperative fluid volumes administered to children receiving kidney transplants. Both fluid volume and urine output were negatively associated with short-term graft function. These data contrast traditional interpretation of high urine output as a marker of graft health, and highlight the need for prospective clinical trials to optimise perioperative fluid administration for this group.

**Graphical Abstract:**

A higher resolution version of the Graphical abstract is available as Supplementary information

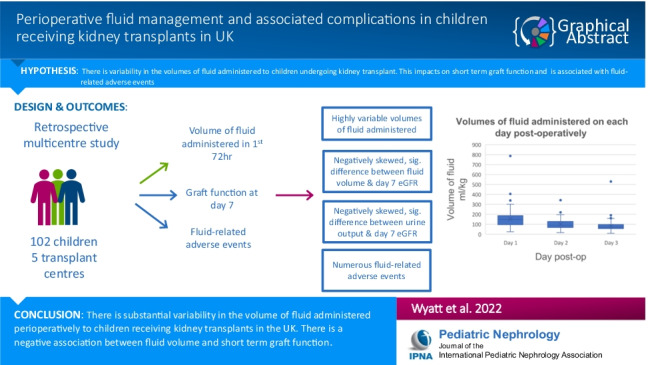

**Supplementary Information:**

The online version contains supplementary material available at 10.1007/s00467-022-05690-3.

## Introduction 

Kidney transplantation is the treatment of choice for children with kidney failure [1]. Kidney transplant innovations have improved the outcomes in children by addressing many factors such as immunosuppression, HLA matching [2], and controlling ischaemia time [3]. However, other modifiable factors have not received as much attention in clinical research. Fluid management is one such factor. In adult transplant recipients, judicious use of fluids was found to be beneficial [4, 5], but there is a high degree of variability in fluid management strategies used, with no consensus on the most advantageous approach [6]. There is also a paucity of high-quality evidence to guide optimal perioperative fluid management in children receiving kidney transplants.

Ensuring adequate perfusion of a transplant kidney is important as intravascular volume depletion brings risk of delayed graft function and acute tubular injury. Delayed graft function in adult recipients is associated with reduced graft survival [7]. Whilst delayed graft function is multifactorial in aetiology, it is exacerbated by poor perfusion with resultant acute tubular injury or kidney arterial thrombosis. The denervation of a graft kidney in the process of transplantation hampers its capacity to regulate blood flow, and as a result, ischaemia and functional impairment may ensue more easily in response to poor perfusion when compared to healthy native kidneys [8].

Whilst inadequate fluid administration may impair graft perfusion, superfluous fluid intake increases the risk of fluid overload with consequential respiratory compromise and hypertension. Respiratory compromise may result in unexpected paediatric intensive care admission and the need for ventilatory support. Systemic hypertension may result in end-organ damage to sensitive vascular beds in the brain and lungs for example. It lies with the healthcare professional team to determine the appropriate volume of fluid to administer to children following kidney transplant.

Current fluid management practices in the immediate postoperative phase following kidney transplantation in children have not been described in the literature. Differing strategies are known to be used, including insensible losses plus ml for ml replacement of urine output and fixed target fluid administration. This study aims to describe the volumes of fluid administered perioperatively across 5 large UK paediatric kidney transplant centres and explore if there are associations between the fluid volume administered with overall graft health or the incidence of adverse events including pulmonary oedema, hypertension, and requirement for intensive care support.

## Method

### Study design

Anonymised perioperative data for children under 18 years of age transplanted between 1 January 2020 and 31 December 2021 in five UK paediatric nephrology transplant centres was collated retrospectively. Those with incomplete data regarding fluid intake and urine output in the first 3 postoperative days were excluded from the analysis.

Data were fully anonymised at individual hospital sites and ethical principles adhered to throughout. The study received local ethical approval, and the requirement for individual participant consent was waived.

### Study procedures

Participants were identified by a lead clinician at each hospital and included all children who had received a kidney transplant between 1^st^ January 2020 and 31^st^ December 2021. Anonymised data were collected by the lead clinician at each hospital via a standardised spreadsheet.

Systemic hypertension was defined as systolic blood pressure above the 95^th^ centile for age and height on two or more consecutive readings [9]. Pulmonary oedema was defined as radiographic evidence of interstitial lung fluid with associated symptoms or signs. Delayed graft function was defined by the requirement for kidney replacement therapy within the first 7 days post-transplant.

### Statistical analysis

Continuous data are summarised as median (interquartile range) or median (range). Non-parametric tests (Wilcoxon’s signed-rank and Mann–Whitney *U*) were used for statistical comparisons. A *P* value < 0.05 was considered statistically significant.

Pre-specified analyses included a comparison of fluid volumes received for children < 20 kg vs ≥ 20 kg bodyweight, relationship between fluid volume administered and day 7 eGFR, relationship between urine output and day 7 eGFR, and comparison of fluid volumes received by children who did and did not experience adverse events (oxygen requirement, pulmonary oedema, systemic hypertension, and unplanned PICU admission). Potential confounders including diuretic dose, use of inotropes, type of transplant, and age were evaluated using non-parametric tests as appropriate.

All statistical analyses were performed using SPSS Statistics for Windows version 28.

## Results

A total of 113 patients were identified from 5 transplant centres. Eleven were excluded due to incomplete data; thus, 102 were included in the final analysis. Forty-two (41%) were female; the median age at transplant was 12.5 (range 2–19; IQR 9) years. Fifty-six (55%) were living donor and 46 (45%) deceased donor transplants. A range of ethnicities were represented (Table 1).

**Table 1:** Descriptive table of demographics overall and differentiated into < 20 kg and ≥ 20 kg groups expressed as number (%). Details are included to describe median total fluid administered, incidence of adverse events, median day 7 eGFR, and median length of stay

Characteristic		< 20 kg group *N* = 28	≥ 20 kg group *N* = 74	Overall *N* = 102
Age at transplant, years Median (IQR)		4 (2)	14 (4)	12.5 ( 9)
Weight, kg Median (IQR)		14.5 (4.5)	45.4 (23)	39.8 (30.9)
Gender *N* (%)	Male	17 (60.7)	43 (58.1)	60 (58.8)
	Female	11 (39.3)	31 (41.9)	42 (41.1)
Ethnicity *N* (%)	White/White British/other White	19 (67.8)	39 (52.7)	58 (56.9)
	Black/Black African/Black Caribbean/other Black	3 (10.7)	6 (8.1)	9 (8.8)
	Mixed ethnicity	0	5 (6.7)	5 (4.9)
	Asian Pakistani/Indian/Bangladeshi	3 (10.7)	18 (24.3)	21 (20.6)
	Other	1 (3.5)	6 (8.1)	7 (6.9)
	Unknown	2 (7.1)	0	2 (2)
Type of transplant *N* (%)	Living donation	20 (71.4)	36 (48.7)	56 (54.9)
	Deceased donation	8 (28.6)	38 (51.3)	46 (45.1)
Underlying diagnosis *N* (%)	CAKUT	13 (46.4)	27 (36)	40 (39.2)
	ARPKD	2 (7.1)	2 (2.7)	4 (3.8)
	Denys–Drash syndrome	0	1 (1.4)	1 (1)
	Vasculitides	1 (3.5)	1 (1.4)	2 (1.9)
	Congenital nephrotic syndrome	3 (10.7)	1 (1.4)	4 (3.8)
	Alport syndrome	0	1 (1.4)	1 (1)
	Manz syndrome	0	1 (1.4)	1 (1)
	Unknown	2 (7.1)	8 (10.8)	10 (9.5)
	Cystinosis	0	1 (1.4)	1 (1)
	Cortical necrosis as a neonate	0	1 (1.4)	1 (1)
	HBNF1B	1 (3.5)	1 (1.4)	2 (1.9)
	PUV	1 (3.5)	14 (18.9)	15 (14.3)
	Nephrotic syndrome	1 (3.5)	4 (5.4)	5 (4.8)
	HUS/TMA	0	2 (2.7)	2 (1.9)
	Nephronophthisis	0	6 (8.1)	6 (5.7)
	Alagille syndrome	1 (3.5)	0	1 (1)
	Joubert syndrome	0	2 (2.7)	2 (1.9)
	Wilms tumour	2	0	2 (1.9)
	Stromme syndrome	1 (3.5)	0	1 (1)
	Primary sclerosing glomerulonephritis	0	1 (1.4)	1 (1)
Total fluid administered ml/kg Median (IQR)		507 (225.5)	356 (139)	374.5 (148)
Occurrence of adverse events N (%)	Pulmonary oedema	3 (10.7)	11 (14.8)	14 (13.7)
	Hypertension	13 (4.6)	40 (54)	53 (52)
	Oxygen requirement	6 (21.4)	25 (33.7)	31 (30.4)
	Unplanned PICU admission	3 (10.7)	6 (8.1)	9 (8.8)
	Delayed graft function	1 (3.5)	2 (2.7)	3 (2.9)
Day 7 eGFR, ml/min/1.73 m^2^ Median (IQR)		135 (60.3)	69.9 (42.7)	84.1 (57.6)
Length of stay Median (IQR)		10 (4)	10 (6)	10 (5)

### Center-specific fluid management guidelines

Each participating center had specific guidelines to assist in the management of children undergoing kidney transplants. Intraoperative fluid management is at the discretion of the operating team. Central venous pressure targets vary between hospitals from 4 to 15 cmH_2_O. Urine output is targeted at > 2 ml/kg/h. All hospitals advocate a fluid plan that includes administration of insensible losses, in addition to the replacement of urine, stool, and other fluid losses millilitre for millilitre. There was variation between hospitals on the calculation for insensible losses with 400 ml/m^2^/day, 20 ml/m^2^/h, and 300 ml/m^2^/day.

### Fluid administered perioperatively

The median total volume of fluid administered, including intraoperatively and the first 72 h posttransplant, was 377 ml/kg (range 122–1184 ml/kg; IQR 149 ml/kg).

#### Fluid administered intraoperatively

**Fig. 1:**
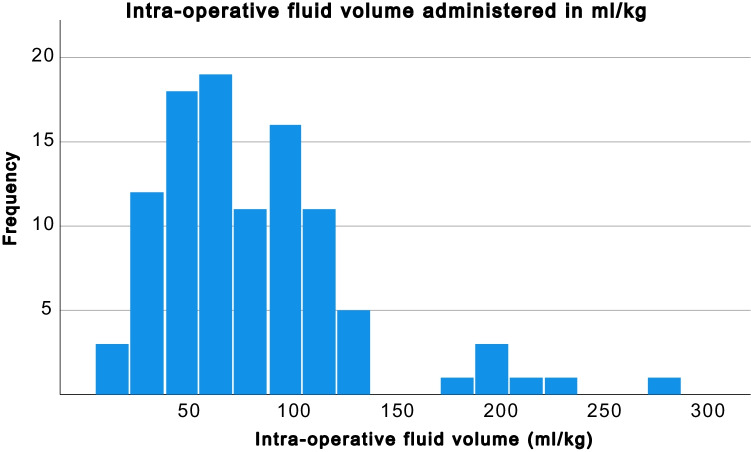
Volume of fluid administered intraoperatively in ml/kg

The median volume of fluid administered intraoperatively was 66 ml/kg (IQR 52 ml/kg) (Fig. 1).

#### Fluid administered in the 3 days postoperatively

**Fig. 2:**
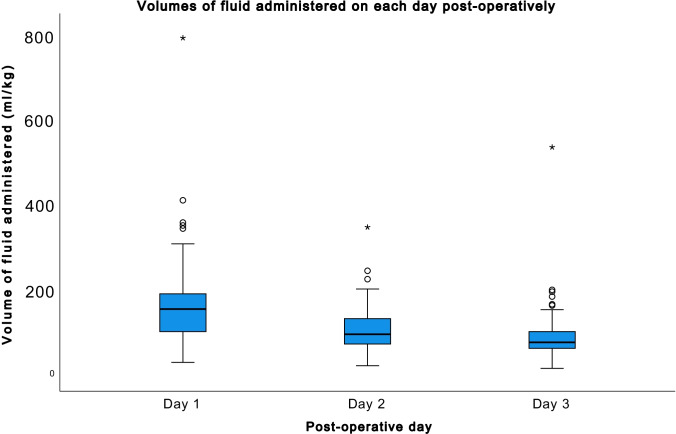
Volume of fluid administered on each of the 3 days postoperatively in ml/kg

The daily fluid volume administered decreased from a median of 148 ml/kg (range 23–786 ml/kg) on day 1 to a median of 70 ml/kg (range 9–194 ml/kg) on day 3 postoperatively (Fig. 2).

### Comparison of fluid volumes administered to smaller (< 20 kg) vs. larger recipients

Children weighing less than 20 kg received a median of 507 ml/kg total fluid volume, compared with 356 ml/kg for children ≥ 20 kg (*p* < 0.001).

Intraoperative fluid volumes were significantly greater for children < 20 kg (median 104 ml/kg vs. 56 ml/kg; *p* < 0.001).

Postoperative fluid administered (excluding intraoperative fluid) was also greater for children under 20 kg (410 ml/kg vs. 294 ml/kg; *p* < 0.001).

### Use of diuretics and inotropic support

Sixty-six (65%) children received furosemide intraoperatively with a median dose administered of 1.5 mg/kg (IQR 1.1). Twenty-nine children (28%) were given mannitol at a median dose of 500 mg/kg (IQR 156). Postoperatively furosemide was given to 39 (38%) children with a median dose of 1 (IQR 1.1) mg/kg.

Inotropes were administered intraoperatively in 68 (67%) children and postoperatively in 39 (38%) children.

### Kidney transplant function

All grafts survived.

**Fig. 3:**
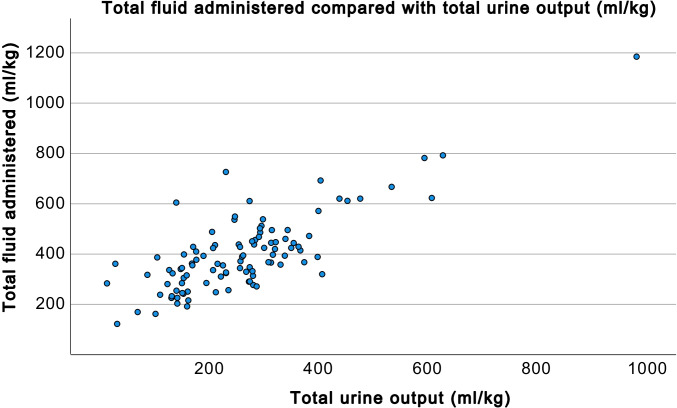
Comparison between total fluid administered and total urine output in ml/kg

The median estimated glomerular filtration rate at day 7 was 84 (IQR 57.6) ml/min/1.73 m^2^. The median total urine output over the whole operative period was 255 ml/kg (IQR 156 ml/kg). The total fluid volume administered exceeded urine output by 131 ml/kg (range − 86 to 496 ml/kg; *p* < 0.001) (Fig. 3). The total diuretic dose was positively related to urine output (*p* < 0.001).

Children weighing < 20 kg had median eGFR 135 ml/min/1.73 m^2^ (IQR 60) at day 7, compared with 70 ml/min/1.73 m^2^ (IQR 42.7) in those ≥ 20 kg (*p* < 0.001).

A negative relationship between total fluid administered and day 7 eGFR was observed (*p* < 0.001); children who received higher fluid volumes had lower day 7 eGFR. A similar negative relationship between the total amount of urine output and the day 7 eGFR was found (*p* < 0.001) (Fig. 5). Neither inotrope use nor demographic factors were significantly related to eGFR. Diuretic dose and transplant type (living vs. deceased donor) associated with eGFR (*p* < 0.001), however, did not explain the negative relationship between eGFR and fluid volume.

Three children experienced delayed graft function. All three received deceased donor kidneys. Two were > 20 kg with an unknown aetiology to their kidney failure; the third was < 20 kg with an underlying diagnosis of CAKUT. None received inotropic support but all three received intraoperative and postoperative diuretics. All experienced hypertension and two were admitted to PICU. All three received less than the median volume of fluid seen in the whole cohort (377 ml/kg) with the < 20 kg child receiving 283 ml/kg in total, and the > 20 kg children receiving 362 ml/kg and 238 ml/kg respectively.

### Adverse events

**Fig. 4:**
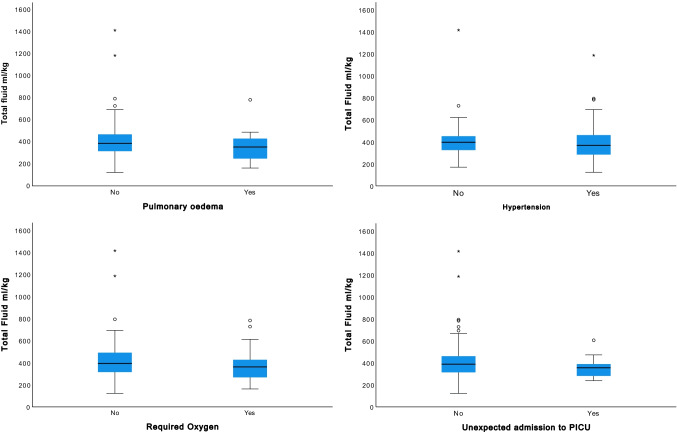
Comparison between the median volumes of fluid administered in total in ml/kg between children who did or did not experience an adverse event during the postoperative period

Systemic hypertension was the predominant adverse event in 53 (52%) children. Oxygen requirement and confirmed pulmonary oedema affected 31 (30%) and 14 (14%) children respectively. Nine (9%) patients had an unexpected admission to paediatric intensive care (PICU) (Table 2). There was no significant difference in the incidence of adverse events in those < 20 kg and those ≥ 20 kg. No significant difference between fluid volumes received in children who did or did not experience these adverse events was found (Table 2, Fig. 4).

**Table 2:** Incidence of adverse events during the postoperative period

Adverse event	Number of patients (%)	Median total amount of fluid received ml/kg in those with the adverse outcome	Median total amount received in the group who did not develop the adverse outcome	Difference in fluid administered and development of an adverse outcome?
Pulmonary oedema	14 (14)	353	386	No (*p* = 0.27)
Hypertension	53 (52)	367	395	No (*p* = 0.28)
Oxygen requirement	31 (30)	361	393	No (*p* = 0.15)
Unexpected PICU admssion	9 (9)	355	388	No (*p* = 0.40)

## Discussion

**Fig. 5:**
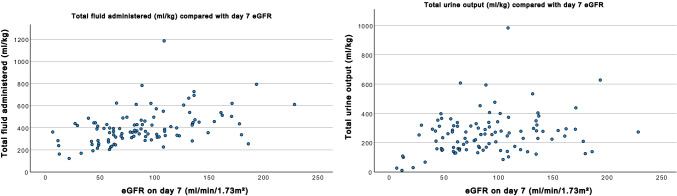
Comparison between day 7 eGFR and total fluid administered in ml/kg, and between day 7 eGFR and urine output in ml/kg

This study shows a high degree of variability in fluid volumes administered to children during and following kidney transplant in current practice. Short-term transplant function was found to be negatively associated with both perioperative fluid volume administered, and volume of urine output (Fig. 5).

In adult kidney transplant recipients, there is a trend for liberal fluid administration with replacement of insensible losses plus supplementary fluid to achieve high central venous pressures and to replace the urine output with the overarching goal of adequate graft perfusion [10, 11]. As such in the adult population, relatively large volumes of intravenous fluid are administered perioperatively to establish and maintain perfusion to the adult donor kidney [12]. Our data suggests that in some cases copious volumes of fluid are given in this paediatric population, but there is substantial variability.

Notably, we have found a significant difference between the total amount of fluid given per kg and day 7 eGFR (*p* < 0.001) with a negative skew, suggesting that increasing volumes of fluid occur with decreasing day 7 eGFR. This observation was not related to diuretic dose or inotrope administration. This result contrasts findings of the 2021 retrospective analysis by Porn-Feldman et al. [13], where it was suggested that a higher intraoperative fluid volume was associated with a lower risk for delayed graft function. In the current retrospective analysis, a causal link between larger fluid volumes and lower short-term graft function cannot be inferred; this negative association may relate to behavioural practice trends—clinicians may respond to impaired transplant function by administering more intravenous fluid.

Urine output is traditionally viewed as a marker of graft function in the postoperative period following kidney transplant [14] and as such fluid may be given in increasing volume, to drive urine output. Loop diuretics may also be administered in order to increase urine output. A large proportion of the cohort (38%) received postoperative furosemide with a positive relationship between the total dose administered and urine output. These data demonstrated a significant difference between the total amount of urine output per kg and the day 7 eGFR with a negative skew. Greater volumes of urine passed are associated with lower eGFR, which suggests that urine output volume may not be a useful marker of short-term graft function as traditionally thought. There are several potential explanations for this observation. Firstly, perioperative administration of loop diuretics can cause a potent rise in urine output which temporarily reduces intravascular volume, thereby impacting graft perfusion and function. Secondly, polyuria can result from ischaemic tubular injury, which is present in varying degrees in all transplant kidneys and reduces GFR [15]. The current study was not designed to differentiate potential mechanisms, and this warrants further prospective evaluation.

All grafts survived in this study. There were three incidences of delayed graft function, all with deceased donor kidneys. Due to the small number of cases of delayed graft function, it was not possible to evaluate potential underlying factors beyond donor type. There were numerous adverse events, notably with systemic hypertension occurring in half the population. A high frequency of hypertension postoperatively has been reported previously in other studies [13, 16]. Whilst the adverse events described in this study are often associated with fluid overload [17, 18] in the current data, there was no significant difference in the median amount of fluid given to those who developed any of the adverse events and those who did not. This is an unexpected result given the increasing recognition by the critical care community that fluid overload is associated with increased morbidity and mortality [19].

The incidence of adverse events was similar in children < 20 kg and those ≥ 20 kg. A significant difference in day 7 graft function was however noted between these groups with smaller recipients having higher eGFR. This was expected due to a greater relative size mismatch of adult donor kidneys in smaller recipients.

This study was limited by its retrospective nature, non-random participant selection due to insufficient data availability in 11 (11%) participants, and restriction to five kidney transplant centres. Notwithstanding these limitations, complete data from a defined 2-year study period on 102 children clearly illustrates the current variation in practice and questions the validity of urine output as a marker of short-term graft health.

In conclusion, there is a substantial variability in the volume of fluid administered to children receiving kidney transplants in current UK practice. A negative association between fluid volume administered and short-term graft function was also observed. It also calls into question the value of using urine output as a key marker of graft function. The retrospective nature of this data precludes the determination of causal relationships, but it does emphatically highlight the need for high-quality prospective clinical trials to optimise perioperative fluid administration in children receiving kidney transplants.

## Supplementary Information

Graphical Abstract (PDF 190 kb)

## Data Availability

Data available upon direct request to the corresponding author.
