# Supplementary material for: Perioperative fluid management and associated complications in children receiving kidney transplants in the UK
Source: Pediatr Nephrol. 2022 Aug 16;38(4):1299–307. doi: 10.1007/s00467-022-05690-3 (PMC9925477; doi:10.1007/s00467-022-05690-3)
Supplement: Supplementary file 1 — (PDF 190 kb) [file 467_2022_5690_MOESM1_ESM.pdf]

Perioperative fluid management and associated complications in children receiving kidney transplants in UK

Graphical Abstract

HYPOTHESIS: There is variability in the volumes of fluid administered to children undergoing kidney transplant. This impacts on short term graft function and is associated with fluid-related adverse events

DESIGN & OUTCOMES:

Retrospective multicentre study

102 children
5 transplant centres

Volume of fluid administered in 1st 72hr

Graft function at day 7

Fluid-related adverse events

Highly variable volumes of fluid administered

Negatively skewed, sig. difference between fluid volume & day 7 eGFR

Negatively skewed, sig. difference between urine output & day 7 eGFR

Numerous fluid-related adverse events

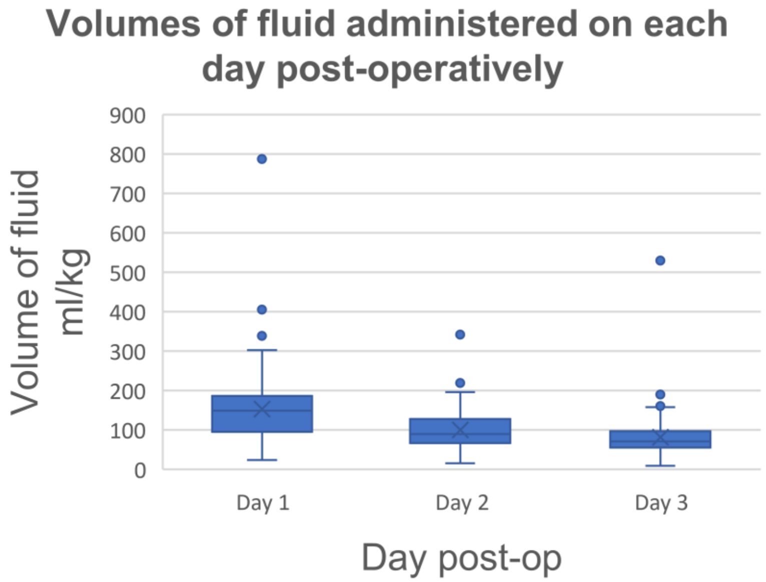

CONCLUSION: There is substantial variability in the volume of fluid administered perioperatively to children receiving kidney transplants in the UK. There is a negative association between fluid volume and short term graft function.

Wyatt et al. 2022

Pediatric Nephrology
Journal of the
IPNA International Pediatric Nephrology Association
